# Mal-positioned Gastrojejunostomy Tube

**DOI:** 10.5811/westjem.2015.9.28562

**Published:** 2015-12-01

**Authors:** Shadi Lahham, Samer Assaf, Romeo Fairley

**Affiliations:** University of California, Irvine, Department of Emergency Medicine, Irvine, California

## CASE REPORT

**Figure 1:**
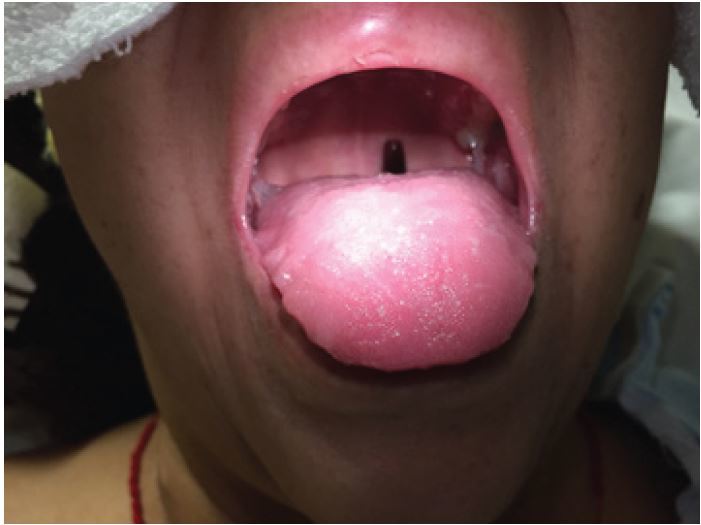
Photograph demonstrating visible gastrojejunostomy tube in patient’s oropharynx.

**Figure 2:**
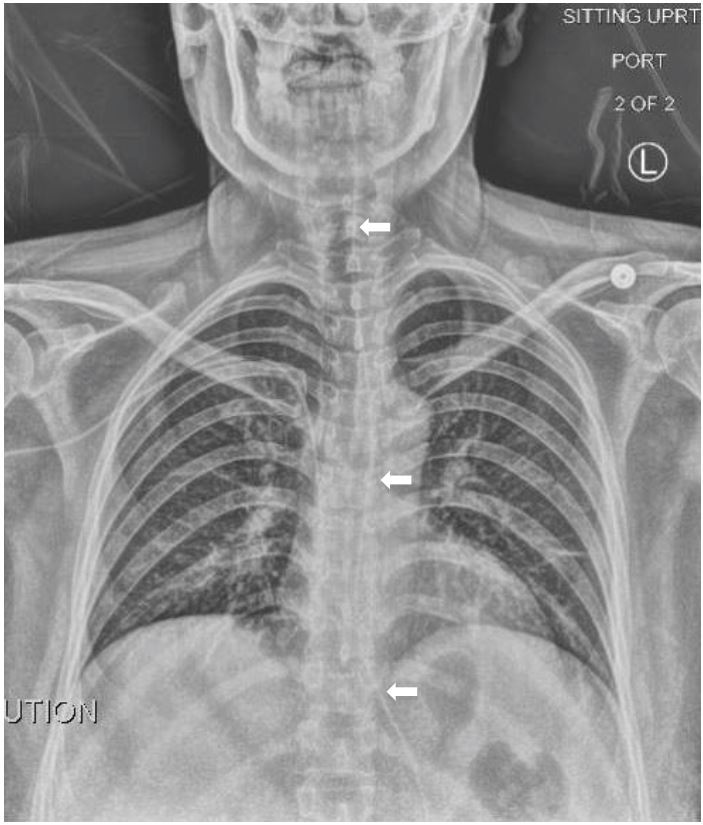
Upright chest radiograph with visible gastrojejunostomy tube superiorly displaced up the esophagus.

A 41-year-old female presented to the emergency department with nausea, vomiting and foreign body sensation in her throat. The patient had multiple co-morbidities including hypertension, diabetes, cervical cancer and gastroparesis with gastrojejunostomy (GJ) tube. The patient had stable vitals, was in no respiratory distress, and her only complaint was mild throat pain and abdominal pain at the GJ tube insertion site. Physical exam revealed a foreign object in the oropharynx (Figure 1). Abdominal exam showed a soft, non-distended, non-tender abdomen with GJ-tube and colostomy in place. Abdominal series and upright chest radiograph were obtained (Figure 2).

## DIAGNOSIS

Mal-positioned GJ tube. Oral exam showed the distal end of the GJ tube protruding into the oropharynx (Figure 1). Upright chest radiograph showed the GJ tube extending superiorly up the esophagus into the oropharynx (Figure 2).

A GJ tube is a percutaneous device that provides access to both the stomach and jejunum^1^,^2^. This tube is positioned at the same location as a gastric feeding tube but is longer in order to reach the jejunum. Its purpose is to provide decompression of the stomach and enteric feeding to patients with poor caloric intake.^3^ The rate of complications of GJ tubes vary between 1–13%.^4^,^5^ Many of these complications are considered minor with <1% causing mortality.^6^–^9^ In patients with vomiting there is a chance that the GJ tube is displaced from the jejunum and can enter into the esophagus. This can be confirmed with chest radiograph or CT chest.^10^,^11^
